# Linking radial growth patterns and moderate‐severity disturbance dynamics in boreal old‐growth forests driven by recurrent insect outbreaks: A tale of opportunities, successes, and failures

**DOI:** 10.1002/ece3.7080

**Published:** 2020-12-14

**Authors:** Maxence Martin, Cornélia Krause, Hubert Morin

**Affiliations:** ^1^ Département des Sciences fondamentales Université du Québec à Chicoutimi Chicoutimi QC Canada; ^2^ Institut de recherche sur les forêts (IRF) Université du Québec en Abitibi‐Témiscamingue Rouyn‐Noranda QC Canada; ^3^ Centre d’étude de la forêt Université du Québec à Montréal Montréal QC Canada

**Keywords:** dendroecology, ecosystem‐based management, forest dynamics, moderate‐severity disturbance, natural disturbance, old‐growth forest, radial growth pattern, spruce budworm (*Choristoneura fumiferana* [Clem.])

## Abstract

In boreal landscapes, emphasis is currently placed on close‐to‐nature management strategies, which aim to maintain the biodiversity and ecosystem services related to old‐growth forests. The success of these strategies, however, depends on an accurate understanding of the dynamics within these forests. While moderate‐severity disturbances have recently been recognized as important drivers of boreal forests, little is known about their effects on stand structure and growth. This study therefore aimed to reconstruct the disturbance and postdisturbance dynamics in boreal old‐growth forests that are driven by recurrent moderate‐severity disturbances. We studied eight primary old‐growth forests in Québec, Canada, that have recorded recurrent and moderately severe spruce budworm (*Choristoneura fumiferana* [Clem.]) outbreaks over the 20th century. We applied an innovative dendrochronological approach based on the combined study of growth patterns and releases to reconstruct stand disturbance and postdisturbance dynamics. We identified nine growth patterns; they represented trees differing in age, size, and canopy layer. These patterns highlighted the ability of suppressed trees to rapidly fill gaps created by moderate‐severity disturbances through a single and significant increase in radial growth and height. Trees that are unable to attain the canopy following the disturbance tend to remain in the lower canopy layers, even if subsequent disturbances create new gaps. This combination of a low stand height typical of boreal forests, periodic disturbances, and rapid canopy closure often resulted in stands constituted mainly of dominant and codominant trees, similar to even‐aged forests. Overall, this study underscored the resistance of boreal old‐growth forests owing to their capacity to withstand repeated moderate‐severity disturbances. Moreover, the combined study of growth patterns and growth release demonstrated the efficacy of such an approach for improving the understanding of the fine‐scale dynamics of natural forests. The results of this research will thus help develop silvicultural practices that approximate the moderate‐severity disturbance dynamics observed in primary and old‐growth boreal forests.

## Graphical abstract

The analysis of radial growth patterns highlights that boreal old‐growth forests are resistant to recurrent and moderate‐severity disturbances, as understory trees can rapidly fill the resulting gaps. However, trees that are unable to attain the canopy following the disturbance then tend to remain in the lower canopy layers, even if a new disturbance reopens the canopy. Therefore, reaching the canopy represents a once‐in‐a‐lifetime opportunity, where success or failure depends on a relatively short window of time.

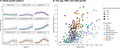

## Résumé

Dans les paysages boréaux, l'accent est désormais mis sur des stratégies de gestion proches de la nature afin de maintenir la biodiversité et les services écosystémiques liés aux vieilles forêts. Le succès de ces stratégies dépend toutefois d'une compréhension fine de la dynamique de ces forêts. Les perturbations de sévérité modérée ont ainsi été récemment reconnues comme étant d’importants moteurs de la dynamique des forêts boréales, mais l’on sait encore peu de choses de leur influence sur la structure et la croissance des peuplements. Par conséquent, l'objectif de cette étude est de reconstruire les dynamiques de perturbation et post‐perturbation dans les vieilles forêts boréales causées par des perturbations récurrentes de sévérité modérée. Nous avons étudié huit vieilles forêts primaires au Québec, Canada, ayant enregistré des épidémies de tordeuse des bourgeons de l'épinette (*Choristoneura fumiferana* [Clem.]) récurrentes et de sévérité modérée au cours du 20ème siècle. Nous avons utilisé une approche dendrochronologique innovante combinant l’étude des patrons et des reprises de croissance pour reconstruire la dynamique de perturbation et post‐perturbation de ces forêts. Nous avons identifié neuf patrons de croissance, observés dans des arbres d'âge, de taille ou de strate de canopée différents, indiquant des dynamiques particulières. Ces patrons ont mis en évidence la capacité des arbres opprimés à rapidement combler les trouées dans la canopée en un unique et significatif accroissement de circonférence et de hauteur. En revanche, les arbres déjà situés dans la canopée ont eu peu d'influence sur la fermeture de ces trouées. En conséquence, les arbres dominants et codominants étaient les plus fréquents dans la canopée. Les résultats de cette étude soulignent la résistance des vieilles forêts boréales aux perturbations récurrentes et de sévérité modérée, car les arbres du sous‐étage peuvent rapidement combler les trouées qui en résultent. Cependant, les arbres incapables d’atteindre le sommet de la canopée à la suite d’une perturbation resteront ensuite souvent dans les strates inférieures de la canopée, même si des perturbations subséquentes créent ensuite de nouvelles trouées. La combinaison de la faible hauteur des arbres typique des forêts boréales, des perturbations périodiques et de la rapide fermeture des trouées forme des peuplements avec une structure verticale ressemblant à celle des forêts équiennes. Globalement, cette étude souligne la résistance des vieilles forêts boréales en raison de leur capacité à supporter des perturbations répétées de sévérité modérée. De plus, l’étude combinée des patrons et des reprises de croissance démontre l’efficacité de cette approche pour reconstruire la dynamique à échelle fine des forêts naturelles. Les résultats de cette recherche contribueront ainsi à développer des pratiques sylvicoles analogues à la dynamique de perturbation de sévérité modérée observée dans les vieilles forêts primaires des paysages boréaux.

## 1 INTRODUCTION

Anthropogenic activities over the last centuries have increased pressure on forest ecosystems, causing a significant loss of natural forest areas (Achard et al., 2009; Aksenov et al., 1999; Watson et al., 2018). Forest artificialization, fragmentation, and deforestation threaten numerous species and ecosystem services, including carbon sequestration and water supply (Karjalainen et al., 2010; Watson et al., 2018). Climate change is expected to increase the frequency and severity of natural disturbances and extreme weather conditions, thereby further stressing forest ecosystems (Gauthier et al., 2015; Jandl et al., 2019; Seidl et al., 2017). To address these issues, researchers have emphasized forest management strategies that aim to mimic natural forest structures and dynamics (Eyvindson et al., 2021; Franklin et al., 2019; Kuuluvainen & Gauthier, 2018). Reducing the difference between managed and natural forests is expected to offset the loss of biodiversity and ecosystem services while increasing the resistance and the resilience of forest ecosystems to climate change (Park et al., 2014; Puettmann et al., 2009).

The desire to reduce differences between managed and natural forests has led to a heightened focus on old‐growth forests—forests in the final stage of forest succession, driven by secondary disturbances (Oliver & Larson, 1996; Wirth et al., 2009). These forests are often the most threatened by human activities, their area greatly reduced through deforestation and intensive forest management (Grondin et al., 2018; Martin et al., 2020; Potapov et al., 2017). Many of the structural attributes that generally define these ecosystems, such as structural and compositional complexity, large trees, and high deadwood volume, are rare, if not absent, in younger or managed forests (Martin et al., 2018; Paillet et al., 2015; Wirth et al., 2009). These structural features provide essential habitats for many species (Boudreault et al., 2018; Tremblay et al., 2018; Winter & Möller, 2008). Similarly, the temporal continuity of old‐growth forests, where the last primary disturbance often occurred centuries ago, is vital for many low‐dispersal (e.g., lichen and bryophyte species) or disturbance‐sensitive species (e.g., woodland caribou (*Rangifer tarandus caribou*)) (Barbé et al., 2017; Faille et al., 2010; Fenton & Bergeron, 2011). Old‐growth forests also play a key role in the offering of ecosystem services, including carbon storage and water flux (Keeton, 2019; Kenina et al., 2019; Warren et al., 2019). Maintaining remnant old‐growth forests or enhancing old‐growth attributes in managed stands has become therefore a common priority for forest and environmental managers (Bauhus et al., 2009; Kuuluvainen, 2009; Thom & Keeton, 2019). The success in achieving conservation objectives related to old‐growth forests depends heavily, however, on fine‐ and multiscale (i.e., at the tree, stand, and landscape scales) knowledge of the dynamics of these ecosystems. Inappropriate management practices, based on superficial knowledge and/or simplification of natural dynamics, may produce limited and even no ecological benefits (Fenton et al., 2013; Puettmann et al., 2009; Venier et al., 2018).

Studies have increasingly highlighted the differences in diversity among stands of old‐growth forests in terms of structure and composition, even within a relatively restricted landscape; this view of old‐growth forests contrasts with the idea of these stands as being homogeneous (Fenton & Bergeron, 2011; Martin et al., 2018; Meigs et al., 2017). Variations in the nature, severity, and recurrence of secondary disturbances play a major role in forming these complex matrices (Martin, Krause et al., 2020; Portier et al., 2018; Svoboda et al., 2014). In particular, recent emphasis has been placed on the importance of moderate‐severity disturbances, also known as intermediate‐severity disturbances, on the dynamics of these ecosystems (Kuuluvainen et al., 2014; Martin et al., 2019; Meigs et al., 2017). Moderate‐severity disturbances are defined as disturbances that exceeds the gap scale (death of one tree or a small group of trees) without being catastrophic (Hart & Kleinman, 2018). Although their existence and importance have been established, knowledge remains nevertheless limited in regard to the consequences of recurrent moderate‐severity disturbances on the structure and dynamics of old‐growth forests. Most research on forest disturbance regimes has generally focused on low‐severity disturbances, that is, gap dynamics, or on catastrophic, stand‐replacing disturbances (Hart & Kleinman, 2018). Many silvicultural treatments considered as "close to nature" are more similar to moderate‐severity disturbances than to gap dynamics, particularly in boreal forests where continuous‐cover forestry practices often harvest a marked proportion (i.e., >30%) of the basal area (Bose et al., 2014; Fenton et al., 2013). In this context, it is therefore important to determine how disturbances of moderate severity influence the structure, resilience, and resistance of old‐growth forests to ensure their sustainable management.

Dendrochronological analysis is an effective tool for reconstructing disturbance dynamics and the associated response of the understory. This reconstruction is usually done by identifying growth releases, that is, abrupt changes in tree‐ring width (Fraver & White, 2005; Nowacki & Abrams, 1997; Trotsiuk et al., 2014). Growth releases nevertheless highlight only punctual changes in growth. They therefore do not make it possible to determine precisely the manner and rate at which a tree grew before and after a disturbance. The study of a tree's growth patterns, that is, the changes in tree‐ring width from the pith to the last formed ring, helps to overcome this problem by making it possible to identify growth releases and also determine how the tree reacted to this release (Martin, Krause, et al., 2020; Trotsiuk et al., 2016). However, the identification of growth patterns has tended to be, at least partially, based on a subjective process, thereby limiting its use (Frelich, 2002; Lorimer & Frelich, 1989; Niukkanen & Kuuluvainen, 2011). Martin, Krause, et al. (2020) highlighted that the use of machine‐learning algorithms provides an effective solution for identifying growth patterns both accurately and objectively. The combined analysis of growth releases and growth patterns therefore offers much promise for reconstructing the dynamics of old‐growth forests driven by natural disturbances.

The boreal forests of eastern Canada offer an ideal territory for addressing questions related to moderate‐severity disturbances because remnant primary forests remain abundant and are dominated by stands at the old‐growth stage (Cyr et al., 2009; Grondin et al., 2018; Watson et al., 2018). In addition, these stands continue to be driven by natural disturbances, in particular by spruce budworm (*Choristoneura fumiferana* [Clem.]) outbreaks, which occur over an approximate 30‐year cycle (Jardon & Morin, 2003; Martin et al., 2019; Morin et al., 2009). Although boreal old‐growth forests are abundant in eastern Canada, they are nonetheless highly threatened by human activities. Forest management based on short‐rotation (70–100 years) clear‐cutting is the main cause of old‐growth forest loss because these stands are harvested first and at rate greater than that of primary disturbances (Barrette et al., 2018; Bergeron et al., 2017; Martin, Boucher, et al., 2020). In the boreal regions, climate change is also expected to increase the recurrence and severity of disturbances, in particular, spruce budworm outbreaks (Bouzidi et al., 2019; De Grandpré et al., 2018; Pureswaran et al., 2019). Hence, a better understanding of the resistance and resilience of boreal old‐growth forests in eastern Canada to recurrent moderate‐severity disturbances is vital to better evaluate the projected consequences of climate change and propose alternatives to clear‐cut‐based forest management. This knowledge would also add to existing research on old‐growth forests and therefore contribute to finding relevant management solutions, including for forests situated outside of the boreal regions of eastern Canada and where natural references are now lacking.

This study focused on the boreal forests of eastern Canada and, more specifically, within a territory subjected to moderate‐severity secondary disturbances caused by spruce budworm outbreaks over the 20th century (Morin & Laprise, 1990; Morin, 1994; Navarro et al., 2018). Innovative dendrochronological analyses, combining the study of growth releases and growth patterns, are used to reconstruct the disturbance regime of the studied stands and evaluate the success of understory and overstory trees in filling the gaps created by secondary disturbances. This study aimed to reconstruct the disturbance and postdisturbance dynamics in boreal old‐growth forests driven by moderate‐severity disturbances. We hypothesize that (a) radial growth in the studied trees can be divided into several distinct growth patterns shaped by the secondary disturbance regime, and (b) each of these growth patterns corresponds to trees defined by specific characteristics (e.g., age, canopy layer), making it possible to reconstruct the postdisturbance dynamics of the forests that they constitute. Our results will contribute to the development of sustainable development strategies that better correspond to the processes driving these ecosystems.

## 2 MATERIALS AND METHODS

### 2.1 Study territory

**Figure 1:**
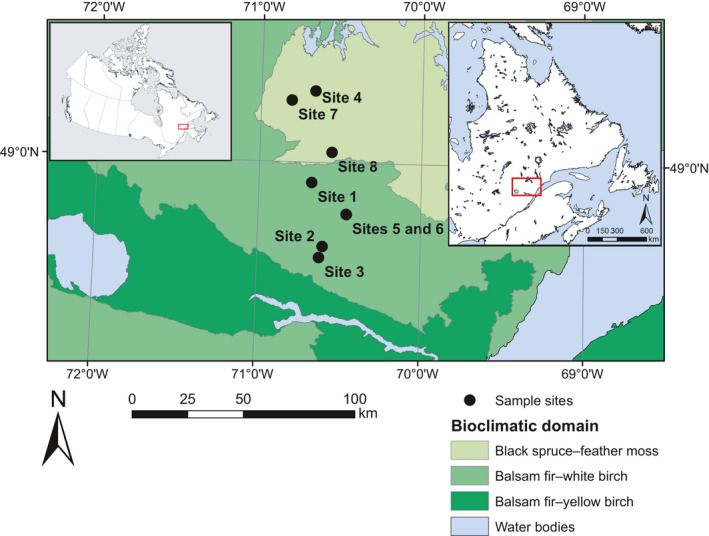
Location of the study sites on the study territory. The inset maps indicate the location of the study territory in Canada (left) and in Québec (right)

Our study took place in the natural boreal forest stands within the Monts‐Valin region of Québec, Canada (Figure 1). The studied area ranges from 48°61′N to 49°30′N and from 70°34′W to 70°82′W in the balsam fir (*Abies balsamea* (L.) Mill.)–white birch (*Betula papyrifera* Marsh.) and the eastern black spruce (*Picea mariana* (Mill.) B.S.P.)–feather moss bioclimatic zones (Saucier et al., 2009). Mean annual temperature, recorded at a weather station located in the study area (Bernatchez station), is between 0.4 and 1.8°C, and mean temperature during the growing season (May–September) is between 12.6 and 13.5°C. Average annual rainfall varies between 886 and 1,109 mm, with an average daily precipitation during the growing season of approximately 2.93 mm/day (Buttò et al., 2020; Rossi et al., 2015). Regional topography is dominated by a hilly relief, and the elevation ranges between 400 and 1,000 m a. s. l. Forest is the main form of vegetation cover across this territory; black spruce, balsam fir, white birch, and aspen (*Populus tremuloides* Michx.) are the most common tree species.

European settlement officially started in the region in 1842, but logging activities were mainly concentrated around inhabited areas and rivers before the second half of the 20th century (Girard & Perron, 1989). In black spruce forests, clear‐cutting was and still is favored because of the small size of the trees. The old‐growth forests still present in the study area can therefore be considered as primary or virgin forests, as no logging activity has directly affected them.

### 2.2 Sampling

We randomly selected eight old‐growth stands across the study territory using a stratified random sampling protocol. We based our sampling criteria on attributes derived from aerial forest survey maps, and these attributes were then verified in the field. We aimed to sample old‐growth forests in this region that had been markedly disturbed by the last spruce budworm outbreak at the time of sampling. The last outbreak occurred between 1972 and 1984 (Krause, 1997; Morin & Laprise, 1990). We initially classified stands characterized as old (i.e., >100 years old), coniferous‐dominated, and defined by a canopy containing at least 20% gaps; we considered this last factor as an indicator of a moderate‐severity disturbance caused by spruce budworm outbreaks, as the stands still present marks of this disturbance several decades later. Signs of past disturbance were then verified in the field. We also confirmed that all study stands were undisturbed by human activities, implying that all the selected sites were primary forests.

Sampling occurred in 2009. In each selected stand, we established a 400‐m^2^ (20 × 20 m) plot within which we surveyed all merchantable trees (diameter at breast height [DBH] ≥9 cm), alive or dead. For each tree, we identified the attributes of species, DBH, height, vitality (alive or dead), and crown status (the stem bearing the apical meristem as either intact or broken). We then felled all merchantable trees within the 400‐m^2^ plots to obtain a more accurate measurement of height and to sample basal disks for subsequent dendrochronological analysis. We only selected basal disks from living coniferous trees, and we rejected any disks marked by substantial amounts of decay that prevented tree‐ring analysis. We obtained 381 basal disks: 290 black spruce and 91 balsam fir. Finally, we also sampled saplings (living trees with a DBH < 9 cm and a height ≥ 1.3 m) in two square 5‐m^2^ plots situated at opposite sides and outside of each 400‐m^2^ plot. For each sapling, we recorded its species, DBH, and height.

For each living tree, we also defined its position in the canopy (hereafter, the “canopy layer,” that is, dominant, codominant, intermediary, and suppressed) following the methodology of the Québec Ministry of Forests, Wildlife and Parks (MRNF, 2008). The canopy layers are based on the dominant height (DH), that is, the mean height of the 100 tallest trees per hectare; therefore, in our case, we used the four tallest trees in each 400‐m^2^ plot. We defined dominant trees as having a height greater or equal to DH, whereas codominant trees have a height less than DH but greater or equal to 2/3 DH. We defined intermediary trees as having a height less than 2/3 DH but greater than 1/2 DH, and suppressed trees have a height less than 1/2 DH.

### 2.3 Data preparation

The 381 basal disks were air‐dried and sanded mechanically in preparation for tree‐ring measurements. We measured tree rings along two radii (radius series) to the nearest 0.01 mm using a manual Henson micrometer (Fred C. Henson, Mission Viejo, CA, USA) or a LINTAB measurement table and TsapWin software (Rinntech, Heidelberg, Germany). We used a combination of visual cross‐dating and the COFECHA computer program (Holmes, 1983) to correct the tree‐ring series. We then obtained a single tree‐ring series for each tree (tree series) by taking the mean value of each tree ring measured in the radius series of the corresponding tree.

To identify a tree's radial growth pattern, that is, the main radial growth trends over time, we used the methods established by Martin, Krause, et al. (2020). Each tree series was divided into 20 segments (20‐segment series), each segment containing a similar number of tree rings—the difference in the number of rings per segment for the same tree never exceeded one ring. The first segment began at the first ring after the pith, and the last segment ended at the last ring produced by the tree. This method allowed us to compare trees of different ages by smoothing interannual growth changes and keeping only the overall trend in radial thickness. The age of sampled trees ranged from 55 to 271 years (mean: 143 ± 39.2 years), and the mean number of tree rings per section was 7.13 ± 2 rings.

For each tree, we also computed the following attributes, hereafter qualified as “growth attributes,” on the basis of the tree series: age, mean tree‐ring width, ring width standard deviation, 5th percentile ring width, and 95th percentile ring width. To reconstruct the disturbance history of the sample sites, we used the methods of Nowacki and Abrams (1997) to identify the annual percentage of growth change (%GC) of the 381 tree series, using the equation:

$$\%\text{GC}=\{\left(M_{2}‐M_{1}\right)/M_{1}\times 100\},$$

where *M*_1_ is the mean ring width for the first 10‐year period, and *M*_2_ is the mean ring width for the subsequent 10‐year period. We defined a major release when %GC ≥ 50%, a minor release when 50% > %GC ≥ 25%, a minor suppression when −25% ≥ %GC > −50%, and a major suppression when %GC was lower or equal to −50%. For each site, we then computed the percentage of trees experiencing a major release, minor release, minor suppression, and major suppression for each year covered by the chronologies. Changes in the annual percentage of growth release between sites were then observed using a locally weighted regression (Trexler & Travis, 1993) and smoothed with a 50% span using the *ggplot* package (Wickham, 2016) in *R* software, version 3.3.1 (R Core Team 2019).

To estimate the succession stage of each study stand, we calculated the cohort basal area proportion (CBAP) of each stand as defined by Kneeshaw and Gauthier (2003), using the methodology of Martin et al. (2018) and Martin, Krause, et al. (2020). CBAP indicates the replacement of the first cohort after the last primary disturbance by successive new cohorts of shade‐tolerant species, and its value ranges between 0 and 1. A CBAP ≈ 0 represents a stand where all trees belong to the first cohort, and a CBAP ≈ 1 represents a stand where the first cohort has almost been entirely replaced by new cohorts, that is, a *true* old‐growth forest sensu Oliver and Larson (1996).

### 2.4 Analysis

To address our hypothesis that radial growth in the studied stands can be divided into several distinct growth patterns, we first identified tree‐ring growth patterns. We used a k‐means clustering algorithm (Hartigan & Wong, 1979) on the 381 20‐segment series by applying the Martin, Krause, et al. (2020) methodology. We based the k‐means clustering on the mean ring width for each of the 20 segments, preliminarily scaled and centered, with each segment considered as a different explanatory variable. To ensure the robustness of the obtained clusters, we performed 1,000 iterations of the k‐means algorithm. We determined the optimal number of clusters (radial growth patterns) using the simple structure index (SSI; Dolnicar, 1999) criterion, with the highest SSI value indicating the optimal k‐means partition.

To address our second hypothesis that each of these growth patterns corresponds to specific layers of the canopy, we then compared the differences in growth attributes, DBH, and height between the growth patterns using mixed‐effect analyses of variance (mixed ANOVA). The fixed effects were the growth attributes, DBH, and tree height; we used sample sites as the random effect. The use of sites as a random variable limited their potential influence (e.g., in terms of fertility) on the size and growth of the trees studied. When necessary, we log‐transformed the data or removed outliers (i.e., values below the 1st percentile and above the 99th percentile) to respect the requirements of mixed ANOVA (i.e., homoskedasticity and the normality of the independent variable for each group). In the case of tree height, we considered broken tree canopies as a possible source of bias because the measured height was, in our case, not the actual tree height. We therefore only considered trees having an intact canopy when comparing tree height between the various growth patterns. When the mixed ANOVA produced significant results, we performed a Tukey post hoc test (Tukey, 1977). The distributions of the species and the canopy layers were also compared between the growth patterns using Fisher's exact test.

For all statistical analyses, we used R software (R Core Team 2019), version 3.3.1, and the *vegan* (Oksanen et al., 2018), *nlme* (Pinheiro et al., 2016), *emmeans* (Russel, 2018), *sjPlot* (Lüdecke, 2020), and *TRADER* (Altman et al., 2014) packages, applying a *p*‐threshold for significance of 0.05.

## 3 RESULTS

### 3.1 Overall stand characteristics

In general, black spruce dominated the sampled stands with a minor (<10%) contribution of balsam fir (Table 1). The exceptions were Site 5, where balsam fir dominated, and Site 8, where both species had a similar abundance. The mean tree age of the stands ranged between 122 and 207 years, and tree age varied considerably within stands, with several trees older than 200 years. CBAP values were mainly equal to 1, indicating stands where no first cohort trees remained. The exception was Site 7, for which the CBAP value was 0.93. Therefore, all studied stands were old‐growth forests, defined by a complex age structure with multiple shade‐tolerant cohorts, including several very old trees (Appendix A). The diametric structure was generally complex, with sapling density 1.75 × to 7.5 × that of trees. Dominant height ranged between 13.6 and 17.1 m, and mean tree height ranged between 9.4 and 12.7 m.

**Table 1:** Structural attributes of the sampled stands

Category	Attribute	Site 1	Site 2	Site 3	Site 4	Site 5	Site 6	Site 7	Site 8
Living trees	Tree density (n/ha^−1^)	1,125.00	1625.00	900.00	1,025.00	1,700.00	1,250.00	1,475.00	616.67
	Sapling density (n/ha^−1^)	4,000.00	4,800.00	5,200.00	6,000.00	3,000.00	5,000.00	6,200.00	4,600.00
Snags	Tree basal area (m^2^/ha^−1^)	16.52	27.50	17.59	20.13	34.19	25.23	24.06	9.98
	Snag density (n/ha^−1^)	75.00	25.00	125.00	175.00	300.00	250.00	125.00	275.00
	Snag basal area (m^2^/ha^−1^)	1.53	0.21	5.44	4.40	5.63	4.00	3.10	5.43
Composition	Black spruce proportion (%)*	94.12	93.87	95.43	93.80	30.76	83.93	93.93	48.37
	Balsam fir proportion (%)*	5.88	6.13	4.57	6.19	69.24	16.07	6.07	49.96
Height	Maximum height (m)	13.64	14.78	15.08	17.60	16.40	17.33	15.54	14.27
	Dominant height (m)	13.64	17.77	15.62	18.00	16.40	16.93	15.53	14.55
	Mean height (m)	9.69	10.20	9.47	12.70	11.20	11.50	11.30	9.36
	Height standard deviation (m)	2.47	2.77	3.01	2.98	3.31	3.26	2.06	2.90
Age structure	Oldest tree age (years)	221.00	246.00	251.00	267.00	177.00	205.00	179.00	206.00
	Mean tree age (years)	159.00	131.00	133.00	207.00	132.00	139.00	133.00	122.00
	Tree age standard deviation (years)	28.20	28.00	40.60	43.20	22.60	31.70	22.60	35.70
	Cohort basal area proportion	1.00	1.00	1.00	1.00	1.00	1.00	0.93	1.00

Note

The asterisks indicate that the value is based on the proportion of the tree species in the tree basal area. Details of age, diameter, and height structure are provided in Appendix A.

### 3.2 Radial growth patterns

**Figure 2:**
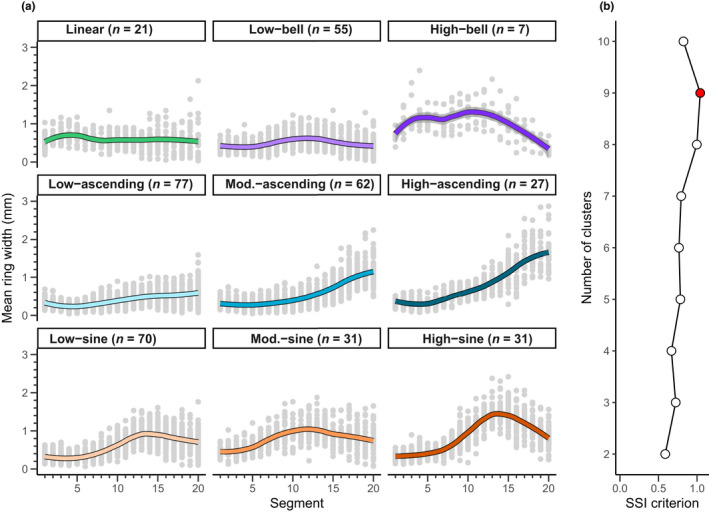
(a) Scatterplots of the growth patterns identified using a k‐means cluster algorithm. Gray dots represent the values of each segment of the 20‐segment series of the trees constituting the clusters. The colored lines represent the loess smoothing of the data with a 50% span. (b) Values of the SSI criterion according to the number of clusters. The red dot indicates the maximum SSI criterion value. Mod.: moderate, *n*: number of sampled trees per cluster

The SSI criterion reached a maximum at nine clusters (SSI criterion = 1.04; Figure 2b) for the 20‐segment series. We therefore divided the 20‐segment series into nine clusters (Figure 2a), which could be, in turn, grouped into four categories: (a) narrow and constant radial increments along all the sections (linear); (b) increasing or large radial increments along the first half of the chronology, followed by decreasing radial increments for the remaining portion (bell); (c) narrow radial increments over the first half of the chronology, followed by increasing radial increments within the remaining portion (ascending); and (d) narrow radial increments over the first third of the chronology, then an increase in radial increments within the second third, and finally a decrease in radial increments over the last third (sine). Growth patterns belonging to the ascending and sine groups were divided into three growth patterns distributed along a mean ring width gradient (narrow, moderate, and large; hence named “low,” “moderate,” and “high”). For the bell group, we identified two growth patterns, one marked by a low growth rate (low‐bell) and the other by a high growth rate (high‐bell). The number of trees per growth pattern generally exceeded 20; the sole exception was for the high bell, a pattern that we only observed for seven trees. Due to the high specificity of this growth pattern relative to the others, however, we kept this pattern for the further analyses.

**Figure 3:**
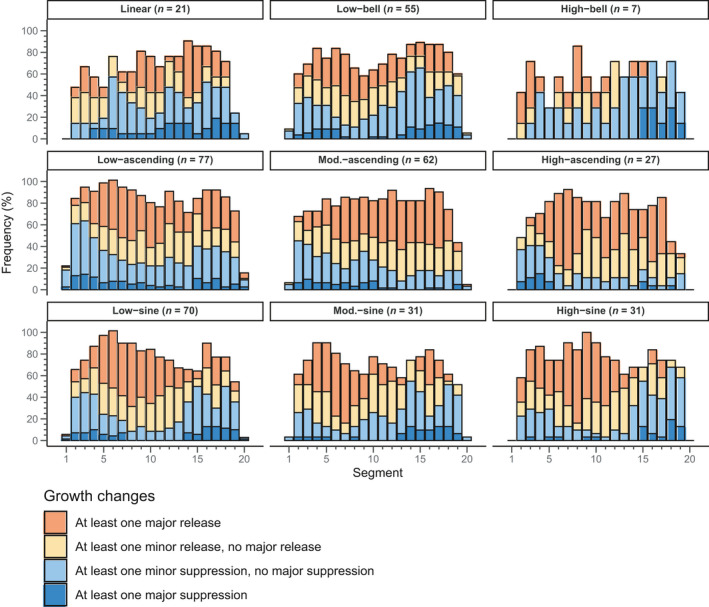
Frequency of growth release and growth suppression observed in the studied trees per growth pattern and section

Growth patterns belonging to the sine and ascending patterns presented mainly growth releases, with a predominance of major releases (Figure 3). Suppressions were less frequent in these patterns, and there were almost no major suppressions. In general, we observed opposite trends for growth release and suppression. For the ascending patterns, suppressions were mainly observed in the first sections, whereas releases were observed in the others. We observed an inverse tendency for the sine patterns, although some suppression could also be seen in the first sections. These results were generally consistent with the growth trends observed for each of these growth patterns. Growth releases and suppressions presented a more complex distribution for the linear, low‐bell, and high‐bell patterns. Growth suppressions were particularly dominant in the high‐bell pattern, even though some growth releases can be observed in the first sections. For the low‐bell patterns, growth releases were generally the most frequent, but they were progressively replaced by growth suppression in the final sections. Finally, the linear pattern presented an alternation between growth release and suppression. These results highlight that low‐bell and linear patterns are defined by complex dynamics. Yet, the slow growth rate of these trees made these variations less marked than those defined by a sine or ascending pattern.

**Figure 4:**
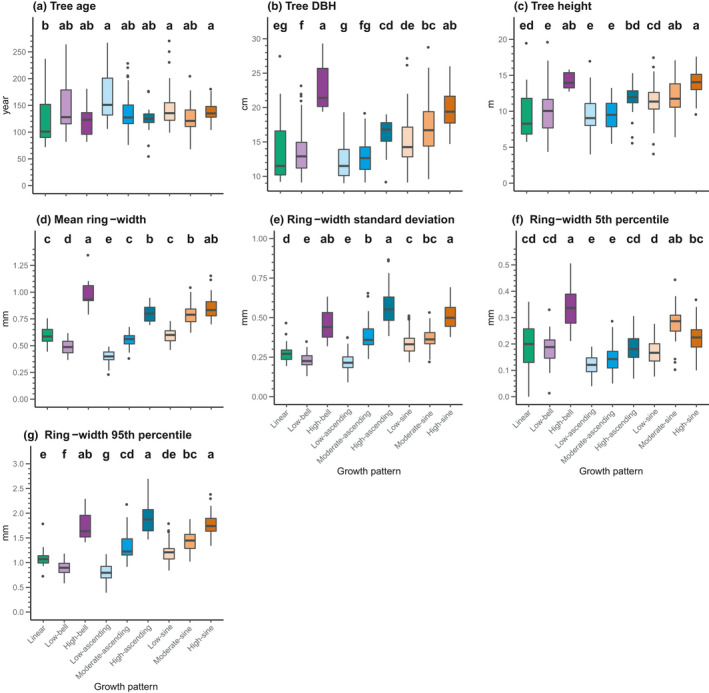
Boxplots of tree and growth attributes per growth pattern. Letters indicate significant differences, with a > b > c > d > e > f > g. Details of the model results are presented in Appendix B. Lines in the boxes represent the median, box boundaries are the 25th and 75th percentiles, vertical lines are values 1.5 × higher or lower than the box boundaries, and the dots represent outliers

**Figure 5:**
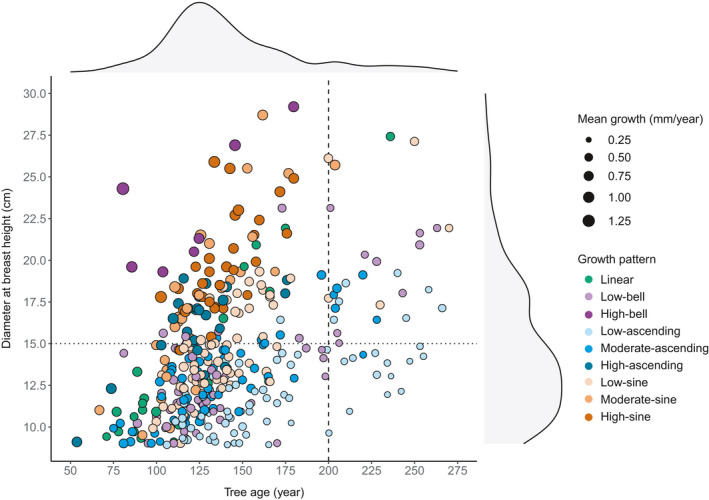
Scatterplot of the studied trees according to their age, DBH, growth pattern, and mean tree‐ring width. Shaded gray areas above and to the right of the scatterplot indicate point density along the *x*‐axis and *y*‐axis, respectively. The dashed line separates trees younger or older than 200 years old; the dotted line separates trees having a DBH greater or less than 15 cm

We observed significant differences between patterns for all growth and tree attributes (Figure 4). There was, however, little difference between the ranges of tree age per growth pattern, although trees defined by low‐ascending, low‐sine, and high‐sine patterns were, on average, significantly older than trees defined by the linear pattern. Trees defined by the high‐bell and high‐sine patterns included the larger and taller trees. In contrast, the linear, low‐bell, low‐ascending, and moderate‐ascending patterns contained the smallest trees, in terms of both DBH and height. High‐bell, high‐ascending, and high‐sine patterns were defined by the largest mean ring width, ring width standard deviation, 5th percentile ring width (high‐ascending excepted), and 95th percentile ring width. In contrast, the low‐ascending pattern was generally characterized by the smallest mean ring width, ring width standard deviation, 5th percentile ring width, and 95th percentile ring width. The other growth patterns presented intermediate results. The largest trees (DBH > 15 cm) were generally defined not only by a mean tree‐ring width > 1 mm/year but also by an age < 200 years (Figure 5). We observed almost all the high‐sine, high‐ascending, high‐bell, and moderate‐sine patterns in these trees. In contrast, trees older than 200 years were uncommon and variable in size and growth, with the low‐ascending pattern being most frequent. Trees younger than 200 years and having a DBH > 15 cm were characterized by diverse growth patterns and variable mean ring widths. Finally, we observed no significant differences in growth pattern occurrence between black spruce and balsam fir (Fisher's *p* = .132; Appendix B).

### 3.3 Distribution of the radial growth patterns in the canopy

**Figure 6:**
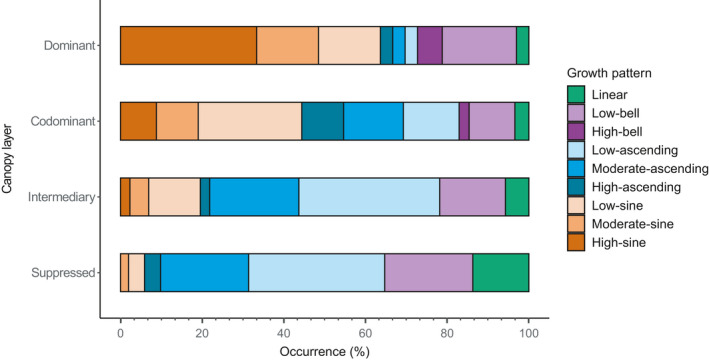
Occurrence (%) of the growth patterns in the different layers of the canopy

The occurrence of specific growth patterns differed significantly between the various canopy layers (Fisher's *p* < .001; Figure 6). In the dominant layers, growth patterns from the sine group were the most common, with the high‐sine pattern being observed the most often, followed by growth patterns from the bell group. In the codominant layer, sine patterns were again the most abundant (with the low‐sine pattern most dominant), but ascending patterns were the second most common group. For the intermediary and suppressed layers, the ascending patterns dominated, particularly the low‐ and moderate‐ascending patterns. In the suppressed layer, however, the low‐bell and linear patterns were the second and third most common patterns, with sine patterns almost absent.

Overall, the codominant layer was the most common canopy layer (53.2 ± 14% of the sampled trees; Table 2). For five of the sample sites (sites 1, 2, 4, 5, and 7), the majority of trees belonged to the codominant layer. At the other sites, the intermediary and suppressed layers contained more than half of the sampled trees. Nonetheless, codominant and dominant trees still represented at least one third of living trees within the stands. Moreover, saplings were always more abundant than trees (Table 1), indicating a dense regeneration in the understory.

**Table 2:** Distribution (%) of canopy layers within the study sites

Canopy layer	Site							
	Site 1	Site 2	Site 3	Site 4	Site 5	Site 6	Site 7	Site 8
Dominant	2.3	3.1	2.3	5.1	3.9	4.1	1.8	3.1
Codominant	59.1	57.8	31.8	66.7	56.9	42.9	69.6	37.5
Intermediary	27.3	20.3	27.3	17.9	19.6	26.5	25.0	31.2
Suppressed	11.4	18.8	38.6	10.3	19.6	26.5	3.6	28.1

### 3.4 Disturbance dynamics

**Figure 7:**
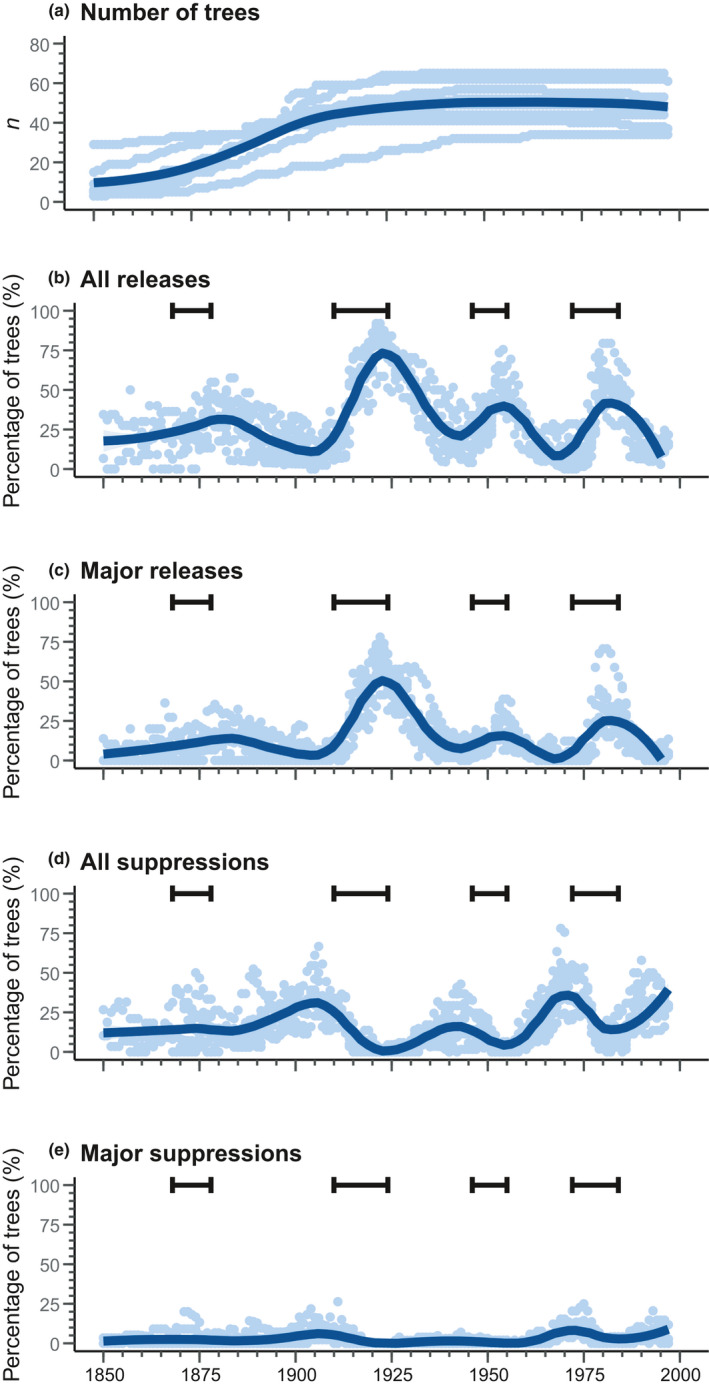
Number of trees sampled and growth changes in the period 1850–2000 for the studied stands (pale blue points). “All releases” includes both minor and major releases; “all suppressions” includes both minor and major suppressions. Dark blue lines represent a loess smoothing of the data with a 50% span. Brackets indicate periods of spruce budworm outbreaks in this region according to Morin and Laprise (1990) and Krause (1997)

At all sites, we observed four distinct peaks of growth release (1870–1890, 1910–1940, 1950–1960, and 1975–1990) between 1850 and 1999 (Figure 7). All peaks occurred during or after a spruce budworm outbreak period and were preceded by a period of suppression. The 1870–1890 peak was the least distinct, characterized by many simultaneously suppressed trees and marked differences in the percentage of released trees between sites. In contrast, the highest and longest peak was that of 1910–1940, when approximately 75% of the trees presented a growth release, two thirds of these were considered as major growth releases. This period was, however, preceded by the second‐highest peak of trees experiencing suppression; these suppressions were nevertheless generally minor. The 1950–1960 and 1975–1990 peaks shared similar percentages of trees undergoing release (approximately 40%); however, most releases for the 1975–1990 peak were major, whereas the majority in the 1950–1960 peak were minor. The 1975–1990 peak was preceded by the highest suppression peak (approximately 35% of trees), whereas the percentage of suppressed trees before the 1950–1960 peak was relatively low (approximately 20% of trees). The study sites therefore appeared mainly driven by recurrent spruce budworm outbreaks, particularly related to a high mortality caused by the outbreak that occurred between 1910 and 1924 in this region.

## 4 DISCUSSION

Tree radial growth was divided into nine distinct growth patterns. These patterns corresponded to four dominant groups that each contained one to three growth patterns, organized generally along a forest productivity gradient. This result supported our first hypothesis, highlighting that secondary disturbances have diverse impacts on overstory tree growth. Canopy layers were each defined by specific radial growth patterns, thereby supporting our second hypothesis. Overall, spruce budworm was the main driver of secondary disturbance within the study sites. Therefore, our study clarifies how secondary disturbance dynamics shape the vertical structure of boreal old‐growth forests that are driven by recurrent and severe insect outbreaks.

### 4.1 Reaching the top of the canopy: a once‐in‐a‐lifetime opportunity

Most of the studied trees were defined by a sine or ascending growth pattern, indicating that black spruce and balsam fir reacted vigorously to the canopy openings caused by spruce budworm outbreaks. Both patterns are generally defined by a major increase in radial growth, a phenomenon observed in coniferous‐dominated old‐growth forests in North America and Europe (Martin, Krause, et al., 2020; Morin, 1994; Trotsiuk et al., 2016). Trees defined by a linear pattern were generally the smaller and younger individuals, reflecting suppressed trees that did not benefit from a canopy opening (Rossi et al., 2009; Trotsiuk et al., 2016) or that were unable to sufficiently increase their growth following gap creation. Trees defined by a low‐bell pattern were similar in size to those presenting a linear pattern albeit slightly older than the low‐bell‐pattern individuals. The minimal and short‐duration increases in radial growth observed for this pattern also related to trees failing to significantly increase their growth following a disturbance. Finally, the high‐bell pattern was highly specific, characterized by a marked growth and no identified juvenile suppression. This pattern was, however, very rare (7 occurrences within 381 studied trees), testifying to its limited influence on stand dynamics and structure.

The identified growth patterns were separated among the various canopy layers. Sine patterns were observed most commonly in the dominant and codominant layers, whereas ascending patterns were most frequent in the intermediary and suppressed layers. This distribution of patterns implies that trees defined by sine patterns had reached the top of the canopy already for several years, whereas trees sharing the ascending pattern continue to attempt to attain the highest layers. Considering the size and the age of the trees, those defined by the high‐ascending or moderate‐ascending patterns were likely to eventually reach the dominant and codominant layers. However, trees in the lower canopy layers and defined by slow growth were unlikely to benefit from the death of taller trees to access the upper layers (Montoro Girona et al., 2016). Therefore, trees defined by a low‐ascending or low‐bell pattern had missed out on the opportunity of reaching the top of the canopy; these trees remain as part of the intermediary or suppressed layers.

Identifying the factors that determine a tree's success or failure in reaching the upper canopy is challenging because these factors are dependent on conditions at the tree, stand, and disturbance levels. For example, local variations in microsite quality significantly influence regeneration growth and density (Jayen et al., 2017; Leroy et al., 2016). Similarly, the spatial patterns of mortality caused by secondary disturbances are often complex, leaving survivor trees in the canopy (De Grandpré et al., 2018; Hart & Kleinman, 2018; Hytteborn & Verwijst, 2014) that may compete with understory trees. We also observed little difference in species composition between the growth patterns, testifying to the similar behaviors of black spruce and balsam fir regeneration under moderate secondary disturbances. Our results therefore highlight that black spruce and balsam fir regeneration can significantly increase their growth after a secondary disturbance and rapidly fill the created gaps. Nonetheless, the time window for attaining the upper layers of the canopy is short, and trees unable to grow sufficiently fast are generally confined to the lower layers. In general, the dynamics observed in this study differ slightly from those identified in broad‐leaved or mixed temperate forests, where growth patterns can be more complex; for example, trees may access the canopy over several steps (Frelich, 2002; Lorimer & Frelich, 1989; Nowacki & Abrams, 1997). Our results illustrate how moderate‐severity disturbances are a once‐in‐a‐lifetime opportunity for suppressed coniferous trees to access the top of the canopy in boreal old‐growth forests.

### 4.2 Recurrent moderate‐severity disturbances are an intrinsic part of old‐growth forest dynamics

The impact of spruce budworm outbreaks on boreal landscapes is highly heterogeneous, ranging from the punctual death of isolated trees to the death of large and continuous forest areas (Kneeshaw et al., 2009; Kulha et al., 2019; Morin et al., 2009). Hence, some regions are marked by significantly higher mortality rates than other areas for a given outbreak (Navarro et al., 2018; Pureswaran et al., 2015). The three 20th‐century spruce budworm outbreaks that occurred within the study territory are characterized by their severity (Morin & Laprise, 1990; Morin, 1994; Navarro et al., 2018), which produced recurrent and significant mortality in the study stands. The peaks in the percentage of trees undergoing release following these outbreaks ranged between 50% and 75% across the study territory, with 20%–55% of the trees presenting major releases. In contrast, for the same outbreaks, old‐growth forests situated approximately 150 km to the north experienced a percentage of trees in release between 20% and 40%, with 15%–25% of these trees presenting a major release (Martin et al., 2019). Martin et al. (2019) determined therefore that true old‐growth forests in this territory were driven almost equally by low‐severity and moderate‐severity secondary disturbances. In contrast, spruce budworm outbreaks in our study territory were twice as severe as the outbreaks documented by Martin et al. (2019), suggesting that all our study stands were driven primarily by secondary disturbances of moderate severity (Hart & Kleinman, 2018; Martin et al., 2019). In some cases, recurrent and severe secondary disturbances may override stand resistance, thereby reinitiating forest succession (De Grandpré et al., 2018; Donato et al., 2012; Meigs et al., 2017). Yet, the structural attributes of the study stands matched those observed in other boreal old‐growth forests in eastern Canada (Harper et al., 2005; Martin et al., 2018; Moussaoui et al., 2019). This implies that the forests observed in this study maintained an old‐growth structure despite the presence of a stressful disturbance regime. Approximately 30 years separated the outbreaks, a period that corresponds to the dynamics and periodicity of this insect in eastern Canada (Morin et al., 2009). Therefore, it suggests that boreal forests in this region are sufficiently resistant to recurrent, moderate‐severity disturbances if these events are sufficiently spaced apart in time.

### 4.3 Complex disturbance and growth processes produced simple vertical structures

Codominant and dominant trees were generally the most common layers in the canopy of the study stands, although the density of saplings was often high. A vertical structure characterized by a majority of dominant and codominant trees is expected in even‐aged forests, whereas old‐growth forests should normally be defined by a complex, multilayered vertical structure (Franklin et al., 2002; Oliver & Larson, 1996). Our results showing a simpler vertical structure matched those of Martin, Fenton et al., (2020), which showed that the vertical structure of dense black spruce‐dominated old‐growth forests was often similar to that of even‐aged stands.

A factor that may explain this counterintuitive result is the low height of boreal stands, which limits the development of complex, stratified vertical structures, such as those observed in temperate and tropical forests (Bergeron & Harper, 2009). In addition, the government of Québec established the height threshold between regeneration and canopy layers at 7 m for Québec (MRNF, 2008). The dominant height of the study stands varied, however, between 13 and 18 m, implying that suppressed trees had maximum heights ranging between 6.5 and 9 m. It is therefore likely that trees below this height threshold were generally too small to be considered as merchantable trees (i.e., DBH ≥ 9 cm) and were therefore classified as regeneration (saplings). Because of the low height and small diameter of boreal trees, the thresholds used to define the different layers may overrepresent codominant trees. In contrast, most suppressed trees are ignored because they do not attain a sufficient height and/or DBH.

Moreover, black spruce regenerates mainly through layering in old‐growth forests (Harvey et al., 2002). Martin, Montoro Girona et al., (2020) hypothesized that these layers are under the hormonal control of the mother tree; the process of apical dominance can inhibit layers' vertical growth because they are still partially connected to the mother tree as branches. Therefore, the death of the mother tree is often required for layers to increase their vertical growth. Similar to black spruce, balsam fir can regenerate by layers in old‐growth forests (Bakuzis & Hansen, 1965; Krause, 2006; Sirois, 1997), suggesting, a priori, similar dynamics. Knowledge related to the importance of layering in balsam fir regeneration remains fragmentary and requires further research. Balsam fir also regenerates by seed under its own cover (Greene et al., 1999; Harvey et al., 2002; Rossi et al., 2012). Balsam fir seedlings can remain suppressed for decades (McCarthy & Weetman, 2006; Morin & Gagnon, 1991; Parent et al., 2000), and their growth rapidly increases only once a gap is created in the canopy (Morin, 1994; Wilson & MacLean, 2015). Generally, the smallest black spruce and balsam fir seedlings present the greatest increases in growth (Martin et al., 2019; Parent & Ruel, 2002). As indicated by our results, the accession to the canopy is therefore often made in one major step rather than over several moderate ones. As a result, old‐growth forests in this region are often defined by dense regeneration layers that increase minimally in height as long as the canopy is not disturbed (Martin, Montoro Girona, et al., 2020).

Finally, spruce budworm outbreaks were periodic disturbances that caused phases of regular and significant mortality in the study stands. This secondary disturbance regime is therefore different from that regularly observed in old‐growth forests, generally defined by a background noise of small‐scale mortality punctuated at random by more severe disturbances (Hart & Kleinman, 2018; Kuuluvainen et al., 2014; Trotsiuk et al., 2014). For this reason, Shorohova et al. (2011) hypothesized that many boreal old‐growth forests of eastern Canada are defined by cohort dynamics, that is, regeneration of new cohorts under the cover of old cohorts due to moderate‐severity disturbances (Angelstam & Kuuluvainen, 2004), similar to Scots pine (*Pinus sylvestris* L.) stands in Fennoscandia or Russia (Angelstam & Kuuluvainen, 2004; Shorohova et al., 2009). Most studied trees presented evidence of juvenile suppression, implying that most seeds or layers appeared before the disturbances. Spruce budworm outbreaks are “top‐to‐down” disturbances, which kill trees in the canopy but preserve some regeneration (De Grandpré et al., 2018; Lavoie et al., 2019). Therefore, in coniferous forests recently disturbed by spruce budworm outbreaks, most of the observed regeneration germinated decades before the disturbance and not immediately after (Martin et al., 2019; Parent et al., 2003; Rossi & Morin, 2011). We therefore consider that the dynamics observed in the study stands likely do not correspond to cohort dynamics. We suggest rather that the dynamics of these forests result from the combination of several interdependent processes: (a) a slow vertical growth of the understory as long as the canopy is not disturbed; (b) a disturbance regime driven by periodic and moderate‐severity disturbances that preserve the regeneration layer; (c) after a disturbance, understory trees reaching the canopy in one major increase in height and diameter; and (d) once the canopy is attained, the trees changing minimally in height.

## 5 CONCLUSION

This study underscored the resistance of boreal old‐growth forests to recurrent moderate‐severity disturbances. Three notable insect outbreaks have marked the history of the studied stands over the last century, with an interval of about 30 years between each outbreak. The growth patterns of the trees indicated that a major portion of the suppressed trees to rapidly reach the upper layers of the canopy and fill the gaps were created following the disturbances. The structure of the stands at the time of the sampling was similar to that commonly observed in primary boreal forests driven by a less severe secondary disturbance regime. Hence, the studied old‐growth forests demonstrated their ability to withstand repeated moderate‐severity disturbances. The combination of low stand height, periodic disturbances, and rapid canopy closure often resulted, however, in relatively simple vertical structures, with a high frequency of dominant and codominant trees. This particularity in the structure of boreal old‐growth forests should therefore be better acknowledged to avoid their misclassification as even‐aged forests in aerial forest surveys.

Several forestry practices have been proposed to maintain old‐growth structures and attributes in boreal landscapes (e.g., partial and stem‐selection cuts). Because of limited tree size and volume, however, foresters generally harvest at least a third of the stand basal area. The effect of such practices can be considered as equivalent moderate‐severity disturbances, with uncertain long‐term impacts on the forests. Our results nevertheless highlight that such silvicultural practices may be close to the natural disturbance dynamics observed in some primary boreal forests. Although complementary research is required, continuous‐cover forest treatments appear as plausible and sustainable alternatives to clear‐cutting in boreal old‐growth forests.

## CONFLICT OF INTEREST

None declared.

## AUTHOR CONTRIBUTIONS

**Maxence Martin:** Conceptualization (lead); data curation (lead); formal analysis (lead); investigation (lead); methodology (lead); software (lead); validation (equal); visualization (lead); writing‐original draft (lead); writing–review and editing (lead). **Cornélia Krause:** Conceptualization (supporting); methodology (supporting); supervision (equal); validation (equal); writing–original draft (supporting); writing–review and editing (supporting). **Hubert Morin:** Conceptualization (supporting); funding acquisition (lead); project administration (lead); resources (lead); validation (supporting); writing–original draft (supporting); writing–review and editing (supporting).

## Data Availability

The data used in this manuscript are available on Dryad (datadryad.org) and are defined by the following https://doi.org/10.5061/dryad.f7m0cfxtq.

## Appendix A

(a) Age structure, (b) diameter structure, and (c) height structure of the study sites.

## Appendix B

Details of the mixed ANOVA

**Table:**

Attribute	*df*	DenDF	*F*‐value	*p*‐value	Transformation
Tree age	8	365	4.18	<0.0001	Log
Mean RW	8	365	163.342	<0.0001	Log
Tree DBH	8	365	32.375	<0.0001	Log
Tree height	8	357	19.897	<0.0001	
RW standard deviation	8	365	86.182	<0.0001	Log
RW 5th percentile	8	361	30.188	<0.0001	Log + outlier removal
RW 95th percentile	8	365	108.065	<0.0001	Log

Abbreviations: DBH, diameter at breast height; DenDF, denominator degrees of freedom; *df*, numerator degrees of freedom; Log, logarithmic; RW, ring width.

## Appendix C

Absolute and relative frequency of the different growth patterns by species

**Table:**

Growth pattern	Species		Total
	Black spruce	Balsam fir	
Linear	13	8	21
	*4.5%*	*8.8%*	*5.5%*
Low‐bell	42	13	55
	*14.5%*	*14.3%*	*14.4%*
High‐bell	4	3	7
	*1.4%*	*3.3%*	*1.8%*
Low‐ascending	54	23	77
	*18.6%*	*25.3%*	*20.2%*
Moderate‐ascending	47	15	62
	*16.2%*	*16.5%*	*16.3%*
High‐ascending	18	9	27
	*6.2%*	*9.9%*	*7.1%*
Low‐sine	60	10	70
	*20.7%*	*11%*	*18.4%*
Moderate‐sine	26	5	31
	*9%*	*5.5%*	*8.1%*
High‐sine	26	5	31
	*9%*	*5.5%*	*8.1%*
Total	290	91	381
	*100%*	*100%*	*100%*

*χ* ^2^ = 12.166 · *df* = 8 · Cramer's *V* = 0.179 · Fisher's *p* = .132

*χ* ^2^, chi‐square, *df*, degrees of freedom.
